# Oral Health of 7- to 9-Year-Old Children Born Prematurely—A Case–Control Observational Study with Randomized Case Selection

**DOI:** 10.3390/dj12120421

**Published:** 2024-12-23

**Authors:** Heide L. Schlesinger, Roswitha Heinrich-Weltzien, Ina M. Schüler

**Affiliations:** 1 Section of Preventive and Pediatric Dentistry, Department of Orthodontics, Jena University Hospital, 07743 Jena, Germany; schlesinger.hl@gmail.com (H.L.S.); roswitha.heinrichweltzien@gmail.com (R.H.-W.); 2 Dental Clinic for Children, Sedation and General Anesthesia, Misgav Ladach Hospital, Jerusalem 9751557, Israel

**Keywords:** dental caries, developmental defects of enamel, prematurity

## Abstract

**Background:** Along with the long-term sequelae of preterm birth for general health, oral health is potentially influenced by prematurity due to developmental and behavioral peculiarities. **Objectives:** This study aimed to compare oral health parameters in the mixed dentition of prematurely and full-term born children. **Methods:** Dental caries, developmental defects of enamel (DDE), and gingival inflammation were assessed in 7-to-9-year-old children (*n* = 38) born preterm (PT) compared to a matched control group born full-term (FT) in Germany. Dental caries was recorded using the International Caries Detection and Assessment System (ICDAS II) and DMFT/dmft-criteria. DDE was scored with modified DDE-Index and periodontal health by Periodontal Screening Index (PSI). Statistical analysis included McNemar’s test and Poisson regression. The significance level was *p* ≤ 0.05. **Results:** Caries prevalence was 47.4% in PT and 57.9% in FT. In the primary dentition, FT children were significantly more affected than PT children (1.6 dmft vs. 2.7 dmft; *p* = 0.035). PT children with extremely low birthweight (ELBW) had the highest caries experience (3.2 dmft; 1.0 DMFT). Prevalence of DDE in primary teeth was significantly higher in PT (55.3%) than in FT children (28.9%; *p* = 0.008). PSI was 3.8 in PT and 3.3 in FT children, but significantly higher in PT children with ELBW (7.4; *p* = 0.125). **Conclusions:** PT children are at higher risk for DDE in primary teeth and compromised periodontal health than FT children. Children with ELBW are most susceptible for dental caries and gingivitis.

## 1. Introduction

According to the World Health Organization (WHO), every birth before 37 completed weeks of gestation is defined as a premature birth [1]. Premature infants are divided into three birthweight groups: 1500 to 2500 g is considered low birth weight (LBW), birth weight between 1000 and 1500 g is considered very low birth weight (VLBW), and birthweight less than 1000 g denotes extremely low birth weight (ELBW). The earlier the pregnancy is interrupted by premature labor, the higher the risk for long-term health impairments [2]. Preterm birth is the main cause of neonatal mortality and poses a risk for long-term physical and neurodevelopmental disabilities [3]. In 2020, globally around 13 million infants were born prematurely, representing 9.9% of all live births in that year [4]. Due to advancements in neonatology, more premature infants survive, even with extremely low birthweight. In Germany, 65% more infants survived premature labor in 2010 compared to 2001 [5]. Advanced maternal age (40 years and over) was clearly associated with preterm birth [6], as well as very young age, low socioeconomic status, low body mass index (BMI), abuse of tobacco and drugs, genito-urinary tract infections, and hypertensive disorders in pregnancy [7]. Periodontal inflammation of the mother also contributed to adverse pregnancy outcomes [8,9]. Further risk factors for premature birth include multiple pregnancies, immune dysregulation, and cervical insufficiency [10].

More than 25% of all premature-born infants suffer from long-term health consequences such as chronic lung diseases, neurological developmental disorders, sensory impairments, and disabilities [3,11,12]. Children born prematurely are more frequently diagnosed with behavioral and socioemotional disorders [13], restrained coordination, and motor skills [14] than mature born children, which might contribute to problems in the provision of daily oral hygiene and oral healthcare at the dentistry office. Further, premature born infants struggle more frequently with feeding difficulties compared to term born infants due to lower muscle tone and impaired coordination of the orofacial structures involved in sucking and swallowing [15], which might favorite the administration of a hypercaloric diet at high frequency and maintaining such cariogenic dietary behaviors.

One of the first oral-health-related observations in PT children was delayed tooth eruption in both dentitions [16,17]. Furthermore, altered tooth dimensions were reported in PT born children [18,19], as well as a higher need for orthodontic interventions [20]. Several studies revealed a higher prevalence of DDE in children born prematurely, especially in the deciduous dentition [21,22].

Enamel formation of deciduous teeth begins in the second trimester and is completed during the first year of life [23]. In healthy infants born FT, the incisal edges and cusp tips of deciduous incisors and molars are fully mineralized at birth [24]. Any insult caused by local trauma, nutrient deficiency, or disturbed cell–cell interaction has the potential to cause a dental defect as dental enamel is incapable of remodeling [25]. It was observed that several systemic diseases and long-term treatment with antibiotics or steroids are associated with developmental defects in both dentitions [26]. Invasive treatments such as intubation or assistance in ventilation in the first months of life contributed to complications in the development of oral tissues [16,27]. Chronic disease of the mother, medication, or drugs and smoking during pregnancy are also associated with a higher prevalence of DDE in PT children [28,29]. DDE are described as qualitative or quantitative alterations of enamel [30], varying between opaque staining, brownish appearance of teeth, or tooth areas up to enamel breakdown and hypoplastic teeth. It seems that cumulative events disturbing enamel formation lead to DDE and that the timing of the insult determines the clinical manifestation [31]. Investigations on exfoliated primary teeth of PT children revealed a different mineral composition resulting in more porous enamel [32].

Findings about DDE in the permanent dentition of PT children are contradictory. One study reported no significant difference in the prevalence of DDE in PT children compared to FT [31], while the prevalence of DDE diagnosed as molar–incisor–hypomineralization (MIH) was significantly higher in cohorts of PT children [33].

A systematic review reported no difference in caries experience between PT and FT children at preschool age [34]. Nevertheless, another study following up children from age 1 to 4 described a higher prevalence of dental caries in children with LBW compared to their peers with normal birthweight [35]. A recent review revealed higher caries experience in the deciduous dentition in children with birth-related complications such as prematurity, born small for gestational age (SGA), or LBW [36]. The review stresses the fact that children with adverse birth outcomes might more often suffer from conditions like DDE, immunodeficiency, or feeding problems and therefore are more susceptible to dental caries. For the association between DDE and dental caries, the term “hypoplasia-associated early childhood caries (HAS-ECC)” was proposed, because the structurally impaired surface of a tooth affected by DDE is particularly prone to being colonized by biofilm and cariogenic bacteria right after eruption [37]. A study examining colonization with Streptococcus mutans in young children stated a higher risk for children with alterations of dental hard tissues [38].

Impaired motor skills of PT children and teenagers [39] might result in poorer oral hygiene, with a higher prevalence of gingivitis and visible plaque in children born PT compared to FT children [33]. Further, PT children and teenagers are a high-risk group for anxiety disorders [40], and dental examination is a common trigger for anxiety in children [41,42]. PT children suffer from higher levels of distress in dental examinations than children born FT [43].

As a contribution to the existing scarce and contradictory literature regarding dental caries and DDE in PT children, this case–control observational study aimed to compare key oral health parameters between PT and FT children in order to state if prematurity constitutes a risk factor for poorer oral health.

The following hypotheses were tested:

1. The prevalence of dental caries in deciduous and permanent teeth is higher in children born PT than in children born FT.
2. The prevalence of DDE in deciduous and permanent teeth is higher in PT children than in FT children.
3. PT children have poorer periodontal health than FT children do.

As every pediatrician and pediatric dentist meets a noticeable number of PT children among their patients, further knowledge about the connection between prematurity and dental health helps to develop effective preventive and treatment strategies for this vulnerable patient group.

## 2. Materials and Methods

This prospective case–control clinical study with randomized case selection was approved by the ethics committee of Jena University Hospital (Reg. Nr.3940/12/13) and registered in the German Registry of Clinical Studies (DRKS00005628). The updated CONSORT Statement 2010 served to guide the reporting.

### 2.1. Study Sample

The study population for this prospective case–control study consisted of 76 children between 7 and 9 years of age. In this age group, deciduous teeth as well as permanent teeth are accessible for examination.

Sample size calculation was based on biostatistical estimation, suggesting a prevalence of 35% for DDE in the targeted age group. To ensure a significance level of α = 0.05 with 80% power, the study group consisting of children born PT required 38 patients.

Patients of the study group were born before 37 weeks of pregnancy between April 2005 and September 2009 and were hospitalized in the Section of Neonatology and Pediatric Intensive Care Medicine of Jena University Hospital. All prematurely born infants are included in the Preterm Registry of the Jena University Hospital.

**Figure 1:**
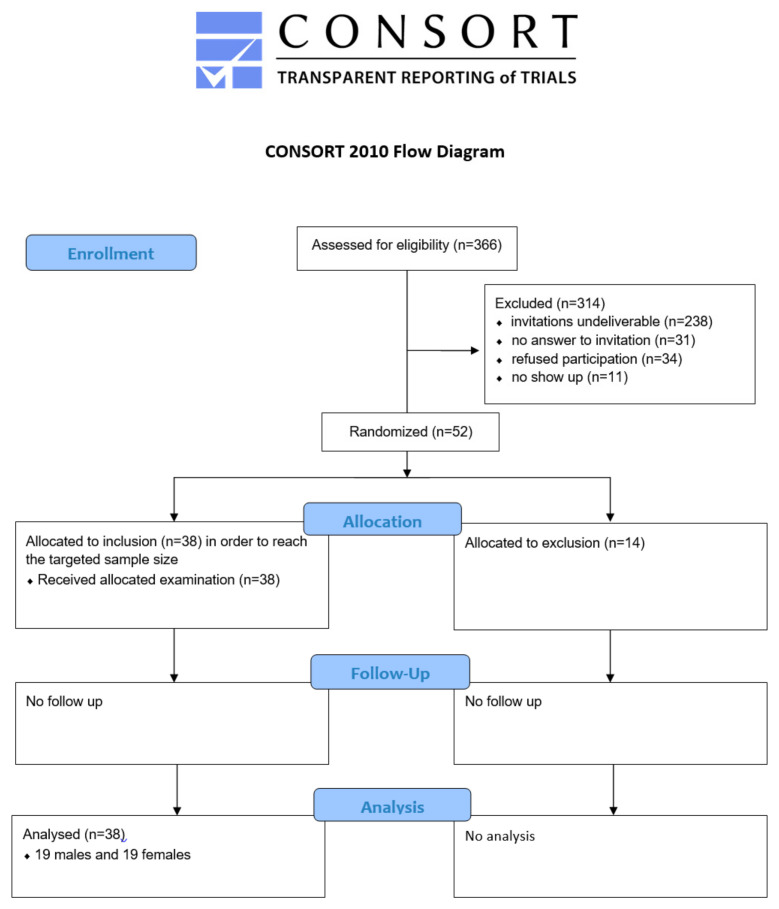
CONSORT flow diagram for selecting patients of the study group.

For the study group, 19 males and 19 females from the Preterm Registry held by the Department of Pediatrics, Unit of Neonatology, Jena University, were selected through a computer-generated randomization list by a statistician. From 366 eligible patients, 238 postal invitations were undeliverable (Figure 1). A further 31 families did not respond to the invitations, and 34 families refused participation due to the long journey (*n* = 21) or too heavy a burden for the child (*n* = 12). One child was deceased. The families were invited by RHW and IMS for a dental examination in the pediatric dental clinic of Jena University Hospital. Eleven children did not show up for the appointment. The remaining patients (*n* = 52) were included in the randomization list.

The study group included three different birth weight groups: 23 were born with low birthweight (LBW: 1500–2500 g), 7 with very low birthweight (VLBW: <1500 g), and 5 with extremely low birthweight (ELBW: <1000 g). Three children in the study group were born prematurely, but with normal birthweight (>2500 g).

The control group of 38 children born on term and above 2500 g was matched by age and sex. The controls were randomly selected from recall patients of the pediatric dental clinic of Jena University Hospital.

Children suffering from genetic or syndrome diseases associated with alterations of dental hard tissues were excluded from the study.

Written informed consent for their children’s participation in the study was obtained from all parents or caregivers. The parents gave permission to obtain medical data from their child’s pediatrician and provided voluntary and anonymous information about their education and employment for their socioeconomic status (SES) to be estimated with the Brandenburg Social Index (BSI) [44]. This index defines high SES for BSI = 9–10, medium SES for BSI = 7–8, and low SES for BSI = 4–6.

### 2.2. Dental Examination

A calibrated dentist conducted oral examinations between April 2014 and September 2016 in the Section of Preventive and Pediatric Dentistry at Jena University Hospital. The κ-values for the intra-rater reliability after calibration resulted in 0.958 (almost perfect) for the detection of caries and 0.775 (substantial) for DDE.

Every patient was examined under standard clinical conditions using a dental mirror and a ball-end probe. No X-rays were taken. After assessment of dental plaque with the Plaque Index (PI) [45] and periodontal health with the Periodontal Screening Index (PSI) [46], all teeth were cleaned with polishing paste and rotating brush. Teeth were dried through air-blowing prior to examination. Dental caries was recorded using the DMFT/dmft-index [47] and the International Caries Detection and Assessment System (ICDAS) II [48]. Developmental defects of enamel were assessed using the modified DDE/dde index [49]. The extent of DDE was categorized in three groups: extent up to one-third of the tooth surface, between one-third and two-thirds of the tooth surface, and more than two-thirds of the tooth surface.

### 2.3. Data Collection and Analysis

The data were recorded in Microsoft Excel 2013, and statistical analysis was performed in Stata/IC 16.0 for Windows. Metric characteristics were described with mean, standard deviation, median, and interquartile range as well as minima and maxima. The 95% confidence intervals were calculated. The Wilcoxon rank-sum test for paired samples was used to test differences in birth parameters and dental parameters between the study group and control group. Spearman’s rank correlation coefficient was calculated to determine correlations between the variables for DMFT, dmft, PI, and PSI. For comparison of the groups regarding prevalence of DDE and dental caries on tooth areas, Poisson regression with random effect was used. Significance level was set at *p* ≤ 0.05.

## 3. Results

### 3.1. Number of Teeth

The average number of permanent teeth was significantly lower in PT than in children born FT. In male children, this difference was higher than in female children but lacked statistical significance. One child born PT had 19 deciduous teeth and no permanent teeth at the age of 7 years, and all other children had an early mixed dentition (Table 1). PT children with ELBW had the lowest number of permanent teeth.

Due to the matching pairs approach, comparisons between PT and FT born children within the different birthweight groups refer to the differences between the children matched by age and gender.

### 3.2. Developmental Defects of Enamel (DDE)

In permanent teeth, the DDE prevalence was higher in PT children compared to FT children. In both groups, females were affected more frequently than males. The prevalence of DDE increased by decreasing birth weight. All differences were without statistical significance (Table 2).

In contrast, the prevalence of DDE in deciduous teeth was significantly higher in PT children than in FT (Table 2). Significantly, more girls born PT had DDE in deciduous teeth compared to girls of the FT group. Within the birthweight groups, children with LBW had the highest prevalence of DDE in deciduous teeth.

The assessment of DDE was based on the examination of 366 permanent teeth in PT children and 405 permanent teeth in FT children. In the PT group, 216 permanent teeth were examined in children born with LBW, 79 in children with VLBW, and 39 in children with ELBW. In the deciduous dentition, 489 primary teeth were examined in PT children and 472 primary teeth in FT children. In the PT group, 297 primary teeth were assessed in children with LBW, 85 in children with VLBW, and 71 in children with ELBW.

Permanent and deciduous teeth revealed predominantly mild manifestations of DDE. Demarcated opacities occurred in 37 (10.1%) permanent and 25 (5.1%) deciduous teeth. Diffuse opacities were diagnosed in 24 (6.6%) permanent and 11 (2.2%) deciduous teeth. Hypoplasia as the most severe manifestation of DDE was detected in 1.6% of both dentitions. Any differences between groups were below the significance level.

DDE covering up to one-third of the surface of the tooth crown was the most frequently found extent in both dentitions (Table S1). In permanent teeth, higher extent occurred predominantly in PT children with VLBW. DDE with extent up to two-thirds and over was diagnosed more frequently in deciduous teeth of males in the PT group. Surprisingly, in males of the FT group, no extended DDE was discovered in deciduous teeth (Table S1).

### 3.3. Dental Caries

#### 3.3.1. Caries Prevalence

The caries prevalence in the mixed dentition was 10% higher in children born FT than in children born PT (57.9%; 95% CI: 40.8–73.7 vs. 47.4%; 95% CI: 31.0–64.2). In the PT group with ELBW, 80% of the children had at least one carious lesion in the mixed dentition, which was the highest prevalence of all groups.

In permanent teeth, the prevalence of initial carious lesions of permanent tooth surfaces (ICDAS II Code 1) did not differ significantly between groups. In children with ELBW, no tooth surface was diagnosed with initial carious lesion, but significantly more tooth surfaces of permanent teeth were affected by more advanced demineralization of the dental enamel (ICDAS Code 2–3), namely, non-cavitated lesions detectable under wet conditions (ICDAS Code 2) and lesions with superficial enamel (micro)cavitation (ICDAS Code 3) compared to children with normal birthweight (PT: 2.4% vs. FT 0.0%).

In primary teeth, the prevalence of initial carious lesions (ICDAS II Code 1) was slightly higher in PT than in FT (1.2% vs. 1.0%). The highest prevalence of initial carious lesions at surface level in the deciduous dentition was observed in children with VLBW and their matching pairs (3.0%). Children with ELBW had significantly more initial carious lesions in deciduous teeth than their matching partners born FT (0.9% vs. 1.4%).

The prevalence of Mod. ICDAS II Code 2–3 carious lesions in deciduous teeth did not differ significantly between groups. The highest prevalence of Mod. ICDAS II Code 2–3 carious lesions was observed in the primary dentition of the children in the ELBW group and their matching partners (FT: 14.2% vs. PT: 10.5%). Children born FT revealed a higher prevalence of manifest caries in deciduous teeth in this study, although the differences lacked statistical significance.

#### 3.3.2. Caries Experience

In the permanent dentition of PT children, a slightly higher caries experience than in FT born children was detected (Table 3). PT born males revealed 0.3 DMFT (SD = 0.7) and 1.6 dmft (SD = 2.3) vs. 0.5 DMFT (SD = 1.1) and 1.5 dmft (SD = 2.2) in PT born females. PT children with ELBW revealed the highest caries experience. In the permanent dentition, PT born males were affected by 0.2 DT (SD = 0.6) and had 0.2 FT (SD = 0.2) compared to PT born females affected by 0.1 DT (SD = 0.3) and 0.4 FT (SD = 1.0). In the deciduous dentition, PT born males revealed a higher dt than females (1.1 dt, SD = 1.6 vs. 0.6 dt, SD = 1.3) and PT born females a higher ft than males (0.9 ft, SD = 1.8) vs. 0.5 ft, SD = 1.5).

Children born FT had significantly higher caries experience in deciduous dentition than children born PT. Children born FT showed significantly more restored deciduous teeth compared to their matching pairs born prematurely. Among PT children, those with ELBW revealed the highest caries experience (Table 3).

### 3.4. Periodontal Health

Dental plaque was detected in 86.6% (*n* = 33) of all PT children, significantly more frequently than in children born FT (63.2%, *n* = 24, *p* = 0.029). PT children had also significantly higher PI scores compared to children born FT. Children with ELBW revealed the highest score for PI (Table 3).

PSI scores per sextant ranged between 0 and 2. The highest PSI per child was 12. Periodontal health issues less affect FT children than PT children (PSI > 0: 57.9% vs. 76.3%). Children born PT revealed slightly higher mean scores for PSI than children born FT, although the difference was not significant. The highest mean PSI was assessed in children with ELBW (Table 3).

### 3.5. Socioeconomic Status

The BSI was used to assess the socioeconomic background of the families. The mean BSI was 8.8 (±1.1, range 5–10) in PT and 9.0 (±1.3, range 5–10) in FT, suggesting a generally high SES of the study population and no significant difference in socioeconomic background of the participants’ families (Table 4). However, the lowest SES was observed in the PT group with ELBW (7.8 ± 0.4; range 7–8).

Comparing the SES of children with and without caries experience revealed that children with caries experience had a lower SES than those who were caries free, regardless of the dentition stage, gender, or premature birth. The difference between children with and without caries experience of approximatively one point causes the shift between the categories high and medium SES, except in the PT groups with VLBW and ELBW (Table 4). In those two groups, also a narrowing of the SES range occurred.

It was further investigated if the extent of caries experience varies by SES. Caries experience in the primary dentition of at least six dmft was associated with a SES lower than eight (7.9 ± 1.7). In the permanent dentition, caries experience of at least two DMFT was associated with a SES lower than eight (7.4 ± 2.1). Highest overall SES was observed in males with caries-free primary teeth (Table 4).

## 4. Discussion

This study is the first comprehensive examination of oral health parameters in the mixed dentition and SES of German school children born PT compared to children born on-term. The results revealed that PT children have an increased risk for DDE in the deciduous dentition and for gingivitis. ELBW poses a higher risk for dental caries, DDE, and gingivitis. Lower SES was associated with lower birth weight and caries experience.

### 4.1. Number of Teeth

In this study, PT children had significantly less permanent teeth than their FT pairs. In the ELBW group, the number of permanent teeth ranged from 0 to 12 (4 first molars, 8 permanent front teeth), wherefrom we can suspect a delayed eruption of the permanent dentition. These findings are consistent with other studies, where delayed eruption in PT children in both dentitions was observed [23,50]. A very recent cohort study also found delayed tooth development to be associated with decreasing birthweight in 7–8-year-old children but did not state statistical significance for this finding [51]. Differences in study designs and missing report on nutritional status, body height, and weight or hormonal and systematic factors [52] might explain the slightly diverging results.

### 4.2. Dental Caries

Infirming the first hypothesis, caries prevalence was higher in FT children compared to PT children, yet without statistical significance. Besides the premature birth, the important role of socioeconomic background during childhood in the development of carious lesions was confirmed [53,54]. In this study, the socioeconomic background of all participating families was predominantly at a high level and similar. Nevertheless, caries experience in either dentition was associated with lower SES compared to caries-free children. The difference of one point in the BSI scale determined the shift between the categories of medium and high SES [44].

While studies suggested a positive correlation between prematurity and dental caries in the deciduous dentition [28,29,35], another study and one systematic review did not confirm this finding [34,55,56]. In this study, the higher caries experience in the deciduous dentition of FT children is the result of a significantly higher number of restored cavities compared to PT children. Children born PT seem to receive dental care less frequently or later in age compared to FT children.

Data regarding the correlation between dental caries in the permanent dentition and LBW in the literature are contradictory. A recent study infirmed such a correlation [57], while another retrospective cohort study reported significantly higher scores for DMFT [58] in PT cohorts. These heterogeneous and insignificant findings might question an isolated and particularly strong effect of prematurity over dental caries development and rather suggest a more complex picture including influences from somatic and psycho-motor development of the child, catch-ups and social settings, and dietary and oral hygiene behaviors. Nevertheless, the higher frequency of endogenic developmental defects of dental hard tissues in premature born children seems to contribute to dental caries development. Teeth with DDE are found to be more vulnerable to dental caries because the altered tooth surface is more prone to plaque accumulation [59]. It was reported that permanent molars with DDE had significantly lower values for enamel hardness and bacterial colonization up to the enamel–dentin junction [60].

### 4.3. Develeopmental Defects of Enamel

The third hypothesis regarding DDE was confirmed in the primary dentition and infirmed in the permanent dentition. This study revealed a significantly higher prevalence of DDE in the deciduous teeth of PT children. In the primary dentition, children with LBW had the highest prevalence of DDE, and children born with ELBW had significantly more tooth areas affected by DDE than children of the FT control group. Compared to the international literature, our findings were higher [61,62,63] but in accordance with the prevalence of DDE in a cohort of German 3–4-year-old children born PT [29]. A recent systematic review confirmed the relationship between prematurity and prevalence of DDE in the deciduous dentition [64].

Contrarily, DDE occurred in permanent teeth, regardless of premature birth. No significant differences between prevalence, severity, or extent of DDE in the PT vs. FT group were detected. Due to delayed eruption of permanent teeth in the PT group, in this study, there were less permanent teeth accessible for assessment than in the case group. Studies including PT and FT school children reported a higher prevalence of DDE in permanent teeth [58] and higher prevalence of MIH [33]. DDE occur during pre- and postnatal enamel formation and maturation, which is very likely compromised by premature labor, interrupted nutrient- and oxygen-supply, and often systematic medication [24], and it can affect any developing tooth. The potential of second primary molars affected by DDE as a possible predictor for MIH in the permanent dentition underpins this etiological explanation as these teeth mature at the same time [65]. A recent prospective study revealed the clear association between PT birth, DDE in deciduous molars, and MIH after following up the patients for 3 years [66].

The question as to whether VLBW or ELBW poses a higher risk for DDE in terms of permanent dentition cannot be answered comprehensively or conclusively based on the data from the literature and our study results. Further investigations with a focus on mother- and child-related risk factors during pregnancy and weaning, epigenetic, or behavioral issues on this topic are needed.

### 4.4. Periodental Health

Significantly higher scores for dental plaque and gingival inflammation in the PT group suggest less effective plaque removal among those children compared to FT children. PT children more often suffer from impaired motor skills and compromised manual development, which can affect the efficacy of manual tooth brushing [33]. Children born with ELBW revealed highest scores for PI and PSI in this study, coinciding with the higher likelihood for impaired motoric development [14]. These findings support the third hypothesis.

### 4.5. Socioeconomic Status

Low parental education level and low family income can coincide with both higher risk for premature birth as well as dental caries and higher prevalence of DDE in deciduous teeth [67].

Although the study population consisted of families with predominantly high and comparable socioeconomic background, the relationship between prematurity, SES, and dental caries development was observed. Children with caries experience in either dentition had a lower SES than those who were caries free, regardless of gender or prematurity. This might suggest that low SES poses a higher risk on dental caries development than prematurity.

### 4.6. Strengths and Limitations

This study focused on the consequences of prematurity in mixed dentition by assessing oral health parameters 7–9 years after premature birth in PT children, while most of the available international studies in PT cohorts focus on younger children. The calibration of the examiner and the calculation of intra-rater reproducibility aimed to reduce observation bias and to increase reliability of the diagnostic results. Efforts were made to reduce the selection bias by randomly selecting PT children from the Preterm Registry and by matching FT children by age and gender. Including matched pairs of children living in a small area of Germany, the homogeneity of the study population strengthens the comparability between groups but reduces concomitantly the external validity and the generalizability. Caries assessment was performed using both ICDAS II- and DMFT-criteria to enable a comparison of study results on an international level.

One of the limitations of this study is the small sample size with regard to the subgroup analysis. Biostatistical estimation of the sample size was targeted at the prevalence of DDE. The further division into three birthweight subgroups resulted in smaller sample sizes per subgroup. Therefore, the subgroup analysis and the analysis of the other oral healthcare parameters might be underpowered, which should be taken into consideration when interpreting and comparing the data. For future studies, the assessment of larger groups is required for more reliable statements on differences in oral health parameters between PT and FT children. Furthermore, general mother- and child-related factors such as chronic diseases of the mother, intake of medication or drugs, smoking, type of delivery, and breastfeeding shall be included in the analysis. These criteria were not taken into account in this study because these data could not be obtained reliably.

## 5. Conclusions

Prematurity constitutes a risk factor for DDE in primary dentition and gingivitis. Children born with ELBW are particularly at risk for DDE, defective periodontal health, and higher caries incidence. Lower SES in PT children was rather associated with higher caries experience in both dentitions than with DDE. Knowledge about this vulnerable risk group might lead to better clinical management of dental problems and help to develop efficient preventative measures for this patient group.

## Figures and Tables

**Table 1:** Number of permanent teeth in the study population (LBW = low birth weight, VLBW = very low birth weight, ELBW = extremely low birth weight; PT = preterm; FT = full-term).

			Number of Permanent Teeth				
	N	Group	Mean	Min-Max	SD	95%. CI	*p* *
All	38	PT	9.6	0–14	2.9	8.7–10.6	0.007
	38	FT	10.6	6–14	1.9	10.2–11.5	
Males	19	PT	9.0	0–12	3.3	7.4–10.6	0.077
	19	FT	10.7	6–14	2.1	9.7–11.8	
Females	19	PT	10.3	6–14	2.4	9.1–11.4	0.031
	19	FT	11.0	8–13	1.7	10.2–11.8	
LBW	23	PT	9.4	4–14	2.7	8.2–10.6	0.010
	23	FT **	11.0	8–14	1.8	10.2–11.8	
VLBW	7	PT	11.3	9–12	1.3	10.1–12.4	0.500
	7	FT **	10.3	6–12	2.2	8.2–12.3	
ELBW	5	PT	7.8	0–12	4.7	1.9–13.7	0.125
	5	FT **	10.6	8–13	2.4	7.6–13.6	

* Wilcoxon rank test for paired data; ** matching pairs, preterms related data are highlighted.

**Table 2:** Prevalence of DDE in the permanent and deciduous dentition according to gender and birthweight groups (LBW = low birth weight, VLBW = very low birth weight, ELBW = extremely low birth weight; PT = preterms; FT = full-terms).

		DDE > 0 in Permanent Teeth				DDE > 0 in Deciduous Teeth			
	Group	N	%	95%-CI	*p* *	N	%	95%-CI	*p* *
All	PT	23	62.2	44.8–77.5	0.346	21	55.3	38.3–71.4	0.008
	FT	19	51.4	34.4–68.1		11	28.9	15.4–45.9	
Males	PT	10	55.6	30.8–78.5	0.739	9	47.4	24.4–71.1	0.414
	FT	9	50.0	26.0–74.0		7	36.8	16.3–61.6	
Females	PT	13	68.4	43.4–87.4	0.317	12	63.2	38.4–83.7	0.005
	FT	10	63.2	38.4–83.7		4	21.1	6.1–45.6	
LBW	PT	14	60.9	38.5–80.3	0.527	13	56.5	34.5–76.8	0.102
	FT **	12	52.2	30.6–73.2		9	39.1	19.7–61.5	
VLBW	PT	5	71.4	29.0–96.3	1.000	3	42.9	9.9–81.6	0.564
	FT **	5	71.4	29.0–96.3		2	28.6	3.7–71.0	
ELBW	PT	3	75.0	19.4–99.4	0.564	2	40.0	5.3–85.3	0.157
	FT **	2	50.0	6.8–93.2		0	0.0	0.0–52.2	

* McNemar Test; ** matching pairs, preterms related data are highlighted.

**Table 3:** Oral health parameters in the permanent and deciduous dentition according to gender and birthweight groups (LBW = low birth weight, VLBW = very low birth weight, ELBW = extremely low birth weight; PT = preterms; FT = full-terms).

Oral Health Parameters	Birthweight Groups			PT	FT
	ELBW	VLBW	LBW		
DMFT mean [95%-CI] SD *p* *	1.0 [−2.2–4.2] 1.0 1.000	0.6 [−0.2–1.3] 0.8 1.000	0.3 [−0.1–0.6] 0.8 0.750	0.4 [0.1–0.7] 0.9	0.3 [−0.0–0.6] 1.0 0.688
dmft mean [95%-CI] SD *p* *	3.2 [−0.4–6.8] 2.9 0.438	1.7 [−0.2–3.6] 2.1 0.313	1.3 [0.4–2.3] 2.2 0.221	1.6 [0.9–2.3] 2.2	2.7 [1.7–3.7] 3.1 0.035
dt mean [95%-CI] SD *p* *	1.6 [−1.3–4.5] 2.3 1.000	1.7 [−0.2–3.6] 2.1 1.000	0.5 [0.1–1.0] 1.0 1.000	0.9 [0.4–1.4] 1.5	0.7 [0.3–1.2] 1.5 0.653
ft mean [95%-CI] SD *p* *	1.4 [−1.8–4.6] 2.6 0.500	0.0 0.0 0.250	0.8 [0.1–1.6] 1.7 0.185	0.7 [0.1–1.2] 1.6	1.8 [1.0–2.7] 2.5 0.009
PSI mean [95%-CI] SD *p* *	7.4 [5.0–9,8] 1.9 0.125	4.6 [2.4–6.7] 2.3 0.438	2.6 [1.5–3.7] 2.6 0.508	3.8 [2.8–4.7] 2.9	3.3 [2.1–4.6] 3.8 0.427
PI mean [95%-CI] SD *p* *	1.3 [0.7–1.9] 0.5 0.188	1.2 [1.0–1.5] 0.3 0.063	0.7 [0.5–1.0] 0.6 0.485	0.9 [0.7–1.1] 0.6	0.6 [0.4–0.8] 0.6 0.027

* Wilcoxon rank test for paired data.

**Table 4:** Socioeconomic status assessed by BSI according to gender preterm birth and birthweight groups, contrasting children with and without caries experience in the permanent (DMFT) and deciduous (dmft) dentition (LBW = low birth weight, VLBW = very low birth weight, ELBW = extremely low birth weight; PT = preterms; FT = full-terms; H = high SES; M = medium SES).

	Socioeconomic Status				
Group (n)	All Mean ± SD Range	DMFT > 0 Mean ± SD Range	DMFT = 0 Mean ± SD Range	dmft > 0 Mean ± SD Range	dmft = 0 Mean ± SD Range
All (76)	H 8.9 ± 1.2 5–10	M 8.2 ± 1.8 5–10	H 9.0 ± 1.0 6–10	M 8.5 ± 1.4 5–10	H 9.3 ± 0.9 7–10
Males (38)	H 9.0 ± 1.3 5–10	M 8.4 ± 1.9 5–10	H 9.1 ± 1.1 6–10	M 8.4 ±1.5 5–10	H 9.6 ±0.7 8–10
Females (38)	H 8.8 ± 1.2 5–10	M 8.0 ± 1.8 6–10	H 9.0 ± 0.9 5–10	M 8.4 ±1.3 5–10	H 9.0 ±1.0 7–10
FT (38)	H 9.0 ± 1.3 5–10	M 8.3 ± 2.1 5–10	H 9.1 ±1.0 6–10	H 8.6 ±1.5 5–10	H 9.6 ± 0.8 7–10
PT (38)	H 8.8 ± 1.1 5–10	M 8.1 ±1.8 5–10	H 8.9 ±0.9 7–10	M 8.4 ±1.3 5–10	H 9.1 ±0.9 8–10
LBW (23)	H 8.8 ± 1.2 5–10	M 7.3 ±2.1 5–9	H 9.1 ±0.8 5–10	M 8.5 ±1.5 5–10	H 9.0 ±0.9 8–10
VLBW (7)	H 9.0 ± 1.3 7–10	H 9.3 ± 1.2 8–10	H 8.8 ± 1.5 7–10	H 9.3 ±1.5 7–10	H 8.8 ± 1.2 8–10
ELBW (5)	M 7.8 ± 0.4 7–8	M 7.0 ± 0.0 7–7	M 8.0 ± 0.0 8–8	M 7.8 ± 0.5 7–8	M 8.0 ± 0.0 8–8

## Data Availability

The raw data supporting the conclusions of this article will be made available by the authors on request.

## Supplementary Materials

The following supporting information can be downloaded at https://www.mdpi.com/article/10.3390/dj12120421/s1. Table S1: Extent of DDE in the permanent and deciduous dentition according to gender and birthweight groups.
