# Supplementary material for: Oral Health of 7- to 9-Year-Old Children Born Prematurely—A Case–Control Observational Study with Randomized Case Selection
Source: Dent J (Basel). 2024 Dec 23;12(12):421. doi: 10.3390/dj12120421 (PMC11674599; doi:10.3390/dj12120421)
Supplement: Supplementary file 1 [file dentistry-12-00421-s001.zip › dentistry-3365930-supplementary.pdf]

Supplemental Material

Table S1. Extent of DDE in the permanent and deciduous dentition according to gender and birthweight groups (LBW=Low Birth Weight, VLBW=Very Low Birth Weight, ELBW=Extreme Low Birth Weight; PT=Preterms; FT=Fullterms)

	Group	N	<1/3 % [95%-CI]	*p**	1/3-2/3 % [95%-CI]	*p**	>2/3 % [95%-CI]	*p**
			**Extent of DDE at crowns of permanent teeth**					
All	PT	366	16.4 [11.2-23.9]	*0.589*	1.4 [0.4-5.1]	*0.287*	0.8[0.2-3.5]	*0.251*
	FT	405	13.8 [8.4-23.9]		3.0 [1.3-6.9]		2.2 [0.4-10.3]	
Males	PT	171	11.1 [6.3-19.2]	*0.291*	2.3 [0.4-10.8]	*0.932*	0.6 [0.1-4.0]	*<0.001*
	FT	196	14.8 [6.8-35.1]		2.0 [0.4-12.8]		0.0-	
Females	**PT**	195	21.0 [13.2-34.3]	*0.210*	0.5 [0.1-3.7]	*0.083*	1.0 [0.1-7.2]	*0.186*
	**FT**	209	12.9 [6.6-25.4]		3.8 [1.5-9.7]		4.3 [0.9-18.9]	
LBW	**PT**	216	14.8 [9.0-25.6]	*0.937*	1.4 [0.3-7.0]	*0.472*	0.9 [0.1-6.4]	*0.190*
	**FT****	252	14.3 [7.8-27.8]		2.8 [0.9-8.4]		3.6 [0.7-15.9]	
VLBW	**PT**	79	20.3 [9.3-41.2]	*0.950*	2.5 [0.4-17.6]	*0.518*	1.3 [0.2-8.8]	*<0,001*
	**FT****	72	19.4 [7.2-52.7]		5.6 [1.3-25.0]		0.0 -	
ELBW	**PT**	39	23.1 [12.0-44.4]	*0.306*	0.0 -	*<0.001*	0.0 -	-
	**FT****	45	13.3 [3.7-45.3]		2.2 [0.3-15.2]		0.0 -	
			**Extent of DDE at crowns of deciduous teeth**					
All	**PT**	489	7.4 [5.0-11.0]	*0.481*	1.4 [0.6-3.2]	*0.271*	0.6 [0.1-4.4]	*0.432*
	**FT**	472	5.9 [3.0-12.6]		0.4 [0.1-3.0]		0.2 [0.0-1.5	
Males	**PT**	247	6.5 [3.1-13.1]	*0.924*	2.4 [1.1-5.6]	*<0.001*	1.2 [0.2-8.7]	*<0.001*
	**FT**	233	6.4 [3.0-15.4]		0.0 -		0.0 -	
Females	**PT**	242	8.3 [5.2-13.6]	*0.349*	0.4 [0.1-3.0]	*0.620*	0.4 [0.1-3.0]	*<0.001*
	**FT**	239	5.4 [1.4-20.2]		0.8 [0.1-6.0		0.4 [0.1-3.0	
LBW	**PT**	297	7.7 [4.6-13.6]	*0.812*	1.3 [0.4-5.2]	*0.580*	0.0 -	*<0.001*
	**FT****	288	8.3 [4.0-18.1]		0.7 [0.1-5.0]		0.3 0.0-2.5]	
VLBW	**PT**	85	5.9 [2.2-15.5]	*0.810*	1.2 [0.2-8.4]	*<0.001*	0.0 -	*<0.001*
	**FT****	86	4.7 [1.0-24.6]		0.0 -		0.0 -	
ELBW	**PT**	71	4.2 [1.4-13.1]	*<0.001*	1.4 [0.2-8.5]	*<0,001*	0.0 -	-
	**FT****	62	0.0 -		0.0 -		0-0-	

*poisson-regression with random effect; **matching pairs, data related to preterms are highlighted.
